# Paradox of trimethylamine-*N*-oxide, the impact of malnutrition on microbiota-derived metabolites and septic patients

**DOI:** 10.1186/s40560-021-00581-5

**Published:** 2021-10-21

**Authors:** Ruey-Hsing Chou, Po-Shan Wu, Shen-Chih Wang, Cheng-Hsueh Wu, Shu-Fen Lu, Ru-Yu Lien, Yi-Lin Tsai, Ya-Wen Lu, Ming-Ren Kuo, Jiun-Yu Guo, Ruey-Yi Chou, Po-Hsun Huang, Shing-Jong Lin

**Affiliations:** 1 Division of Cardiology, Department of Medicine, Taipei Veterans General Hospital, Taipei, Taiwan; 2 Department of Critical Care Medicine, Taipei Veterans General Hospital, 112, No. 201, Sec. 2, Shih-Pai Road, Taipei, Taiwan; 3 Cardiovascular Research Center, National Yang Ming Chiao Tung University, Taipei, Taiwan; 4 Institute of Clinical Medicine, National Yang Ming Chiao Tung University, Taipei, Taiwan; 5 Division of Clinical Nutrition, Department of Dietetics and Nutrition, Taipei Veterans General Hospital, Taipei, Taiwan; 6 Department of Anesthesiology, Taipei Veterans General Hospital, Taipei, Taiwan; 7 Department of Nursing, Taipei Veterans General Hospital, Taipei, Taiwan; 8 School of Nursing, National Yang Ming Chiao Tung University, Taipei, Taiwan; 9 Division of Gastroenterology and Hepatology, Department of Medicine, Taipei Veterans General Hospital, Taipei, Taiwan; 10 Healthcare and Services Center, Taipei Veterans General Hospital, Taipei, Taiwan; 11 Taipei Heart Institute, Taipei Medical University, Taipei, Taiwan; 12 Division of Cardiology, Heart Center, Cheng-Hsin General Hospital, Taipei, Taiwan

**Keywords:** Trimethylamine *N*-oxide, Gut microbiota, Sepsis, Nutrition, Inflammation

## Abstract

**Background:**

Trimethylamine *N*-oxide (TMAO) is a microbiota-derived metabolite, which is linked to vascular inflammation and atherosclerosis in cardiovascular (CV) diseases. But its effect in infectious diseases remains unclear. We conducted a single-center prospective study to investigate association of TMAO with in-hospital mortality in septic patients admitted to an intensive care unit (ICU).

**Methods:**

Totally 95 septic, mechanically ventilated patients were enrolled. Blood samples were obtained within 24 h after ICU admission, and plasma TMAO concentrations were determined. Septic patients were grouped into tertiles according to TMAO concentration. The primary outcome was in-hospital death, which further classified as CV and non-CV death. Besides, we also compared the TMAO concentrations of septic patients with 129 non-septic patients who were admitted for elective coronary angiography (CAG).

**Results:**

Septic patients had significantly lower plasma TMAO levels than did subjects admitted for CAG (1.0 vs. 3.0 μmol/L, *p* < 0.001). Septic patients in the lowest TMAO tertile (< 0.4 μmol/L) had poorer nutrition status and were given longer antibiotic courses before ICU admission. Circulating TMAO levels correlated positively with daily energy intake, the albumin and prealbumin concentration. Compared with those in the highest TMAO tertile, septic patients in the lowest TMAO tertile were at greater risk of non-CV death (hazard ratio 2.51, 95% confidence interval 1.21–5.24, *p* = 0.014). However, TMAO concentration was no longer an independent predictor for non-CV death after adjustment for disease severity and nutritional status.

**Conclusion:**

Plasma TMAO concentration was inversely associated with non-CV death among extremely ill septic patients, which could be characterized as TMAO paradox. For septic patients, the impact of malnutrition reflected by circulating TMAO levels was greater than its pro-inflammatory nature.

**Supplementary Information:**

The online version contains supplementary material available at 10.1186/s40560-021-00581-5.

## Background

Sepsis, a life-threatening disease caused by a dysregulated host response to infection and organ dysfunction [1], is the leading cause of death in intensive care units (ICUs). Emerging evidence suggests that intestinal microbiota imbalances are associated with various inflammatory and metabolic diseases, including atherosclerosis [2], diabetes [3], obesity [4], and dyslipidemia [5], but few studies have examined the roles of microbiota or their metabolites in septic patients [6].

Trimethylamine *N*-oxide (TMAO) is a proinflammatory metabolite that originates from the bacterial metabolism of choline-rich foods, such as red meat and eggs [7]. Accumulating evidence suggests that TMAO is associated with vascular inflammation [8] and atherosclerosis [9]. Elevated plasma TMAO levels have been linked to worsening prognoses in patients with coronary artery disease (CAD) [9], chronic kidney disease [3], and chronic obstructive pulmonary disease [10]. Dietary supplement with choline enhances atherosclerosis in the apoE^−/−^ mice. Elimination of intestinal microbiota by antibiotics reduces plasma TMAO concentration and mitigates its proatherosclerotic effect [11]. However, the role of TMAO in the context of infectious diseases remains unclear. In addition, no clinical data on the impacts of plasma TMAO concentrations in septic patients are currently available.

We conducted this single-center prospective observational study to investigate associations between plasma TMAO concentrations and all-cause mortality in septic patients. Detailed information about participants’ nutritional status and antimicrobial therapy, as well-known confounding factors for TMAO [9], was collected. We hypothesized that higher TMAO levels would be associated with more severe inflammation and worse outcomes in septic patients.

## Methods

### Study populations

This study was approved by the Research Ethics Committee of Taipei Veterans General Hospital (no. 2018-02-009AC) and conducted according to the principles expressed in the Declaration of Helsinki. All participants provided written informed consent. We prospectively screened 116 patients aged > 18 years who were admitted to the medical ICU of Taipei Veterans General Hospital, a tertiary medical center in Taiwan, between September 2018 and January 2020. Patients were admitted to the medical ICU because of various critical illnesses, including acute respiratory failure and hemodynamic instability. Sepsis and septic shock were defined according to the 2016 Surviving Sepsis Campaign guidelines [1]. Patients without sepsis, mechanical ventilation requirement, and the pre-dialysis patients, who are known to have extremely high TMAO concentrations [12], were excluded from this study. Information about enrolled patients’ age, sex, smoking history, comorbidities, nutritional status, infection causes, number and duration of previous antibiotic treatment courses was collected by detailed chart review. Blood cell counts and chemistry parameters were measured at the time of ICU admission. The estimated glomerular filtration rate (eGFR) was calculated using the Chronic Kidney Disease Epidemiology Collaboration equation [13]. Acute Physiology and Chronic Health Evaluation II (APACHE II) [14] and Sequential Organ Failure Assessment (SOFA) scores [15] were calculated within 24 h after ICU admission. We also compared the plasma TMAO concentrations of septic patients with those without sepsis. As the non-sepsis group, we enrolled 129 subjects admitted for elective coronary angiography (CAG) whose plasma TMAO concentrations had been determined in our previous work [16]. Pre-dialysis patients and those with acute myocardial infarction (AMI) or without available C-relative protein data were excluded from this group.

### Measurement of plasma TMAO concentrations

Trained registered nurses obtained blood samples from the enrolled septic patients within 24 h after ICU admission. The blood samples were centrifuged, and 200 μL plasma was mixed with TMAO-d9 isotopologues. After filtration, TMAO concentrations were quantified using a stable isotope dilution assay and high-performance liquid chromatography, with online electrospray ionization tandem mass spectrometry performed on an API 4000 Q-TRAP mass spectrometer (AB SCIEX, Framingham, MA, USA). The septic patients were allocated to tertiles according to their plasma TMAO concentrations.

### Measurement and calculation of nutritional indicators

As traditional nutritional markers [17], serum albumin and prealbumin values were measured by commercial kits (Beckman Coulter, Brea, CA, USA) at the time of ICU admission. For the septic patients admitted to the ICU, detailed information about enteral and parenteral calorie and protein intakes was recorded every 8 h. Daily calorie/protein intake was defined as the average intake per day in the first 48 h of ICU admission. Because indirect calorimetry was currently unavailable in our hospital, we used weight-based equations to determine the energy requirements as the suggestion of guidelines [18, 19]. Target calorie requirements were calculated as 25–30 kcal/kg/day, adjusted by BMI [20]. Target protein requirements were calculated as 1.3 g/kg/day [18].

According to the guideline of American Society for Parenteral and Enteral Nutrition [19], we routinely used Nutrition Risk Screening 2002 (NRS-2002) score to determine the nutrition risk of patients admitted to ICU. NRS-2002 score had been used to access the nutrition risk in critically ill patients in previous studies [21–23]. Patients with NRS-2002 ≥ 5 were considered to be at high risk of malnutrition, and was associated with greater in-hospital mortality [24]. Experienced nursing staff measured the height and weight of the patients, and interviewed them or their family members to identify changes in dietary intake in the previous week, weight loss in the previous 3 months, and the severity of disease [25]. Based on these data, the patients’ body mass index (BMI) and NRS-2002 scores [25] were calculated and documented in their medical records. The nutrition risk index (NRI) was also calculated using the serum albumin and body weight values measured at ICU admission, as 1.519 × albumin (g/L) + 41.7 × (present body weight/ideal body weight) [26, 27].

### Definition of clinical outcomes

The primary outcome was in-hospital all-cause death, classified further as cardiovascular (CV) and non-CV death. Patients who died of AMI, sudden cardiac death, heart failure, stroke, or CV procedures were allocated to the CV death group [28]. Those who died of other causes, primarily sepsis and terminal cancer, were allocated to the non-CV death group. Secondary outcomes included acute kidney injury (AKI) within 48 h after ICU admission, AKI required dialysis during ICU stay, and successful ventilator weaning during hospitalization. AKI was defined according to the Kidney Disease Improving Global Outcomes criteria [29]. Patients who were ventilator independent at discharge were deemed to be weaned successfully [30]. The duration of ventilator use and lengths of ICU and hospital stays were also recorded.

### Statistical analysis

Continuous variables were expressed as medians (interquartile ranges) and analyzed using the Mann–Whitney *U* test or Kruskal–Wallis test. Categorical variables were presented as numbers (percentages) and assessed using Fisher’s exact test or the Chi-squared test. Spearman’s rank correlation test was used to assess correlations between TMAO concentrations and nutritional indicators. Multivariate linear regression analysis was performed to investigate relationships between TMAO values and clinical factors. The incidence of in-hospital, all-cause death (further classified as CV death and non-CV death) and successful ventilator weaning were calculated. Kaplan–Meier analysis and the log-rank test was used to determine the cumulative incidences of death and successful ventilator weaning, stratified by TMAO concentrations. Cox regression analysis was performed to identify predictors of non-CV death and successful ventilator weaning. Variables with *p* < 0.1 in the univariate regression analysis were included in an adjusted forward-stepwise multivariate regression model. *p* Values < 0.05 were regarded as significant. The analyses were performed using SPSS (version 18.0; SPSS Inc., Chicago, IL, USA) and MedCalc (version 11.4.2.0; MedCalc Software, Mariakerke, Belgium).

The sample size was calculated using PASS (version 15.0.5; NCSS, LLC., Kaysville, Utah, USA). The calculation was based on assuming three independent study groups and the primary outcome was in-hospital mortality. As there is currently no data for septic patients grouped by TMAO concentrations, we estimated the sample size by data from the community-acquired pneumonia population [31]. Plasma TMAO concentration was 2.3, 3.0, and 4.1 μmol/L, respectively, in patients with varying mortality rate of community-acquired pneumonia. Using the Kruskal–Wallis test, a total sample of 45 subjects are required to achieve a power of 0.900 with a target significance of 0.050. Considering the prevalence of sepsis was 44% for the critically-ill population in our previous work [32], we need to screen at least 102 patients admitted to ICU to complete this study.

## Results

### TMAO concentrations and nutritional status in septic patients

**Fig. 1:**
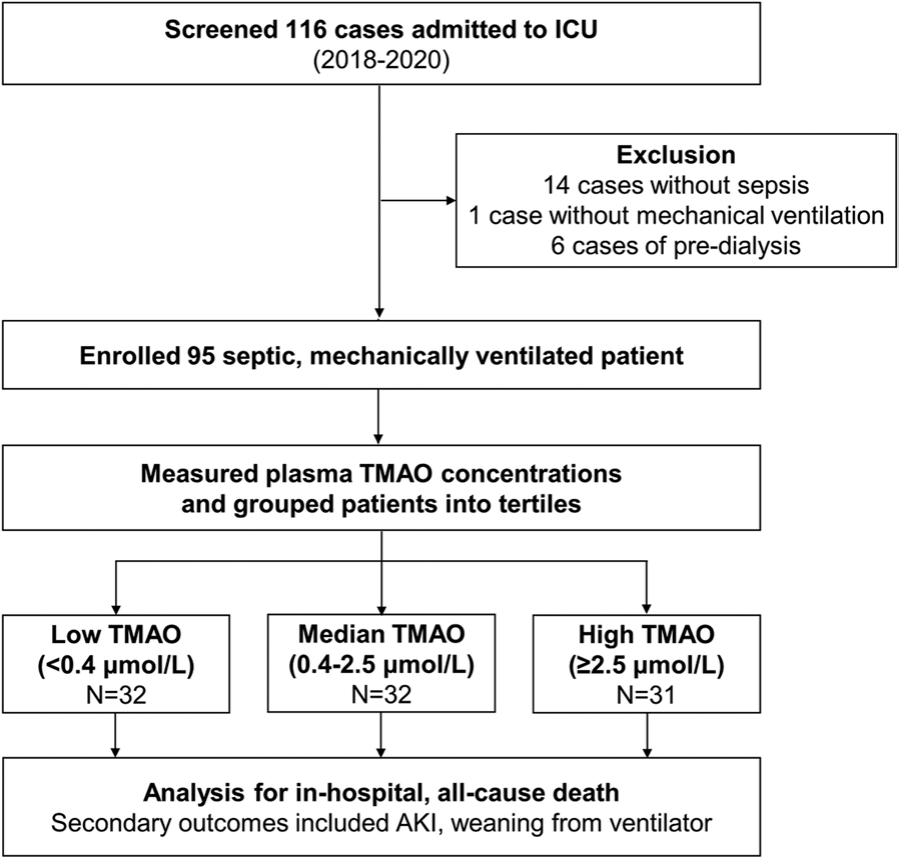
Flowchart of patient enrollment and classification. *ICU* intensive care unit; *TMAO* trimethylamine-*N*-oxide; *AKI* acute kidney injury

One hundred and sixteen patients admitted to ICU during 2018–2020 were screened. After the exclusion of patients without sepsis (*N* = 14) and mechanical ventilation (*N* = 1) and pre-dialysis patients (*N* = 6), 95 septic, mechanically ventilated patients were enrolled in this study. The median age of the septic patients was 70.0 (60.0–78.0) years, and 58 (61.1%) of them were male. Respiratory tract infection was the leading cause of sepsis (80.0%), and 34 (35.8%) patients had septic shock. A flowchart of patient enrollment and classification was provided, as shown in Fig. 1. Age, sex, APACHE II and SOFA scores, and the prevalence of septic shock were similar in the groups defined by TMAO tertiles (Table 1). Patients with high TMAO concentrations (≥ 2.5 μmol/L) had higher prevalence of CAD, stroke, and peripheral arterial disease, and higher C-reactive protein (CRP) levels and lower eGFRs at the time of ICU admission. Compared with patients with high TMAO values, patients with low TMAO concentrations (< 0.4 μmol/L) had been given more and longer antibiotic treatment courses before ICU admission and had lower daily calorie and protein intakes, BMIs, albumin and prealbumin levels, NRIs, and higher NRS-2002 scores. Opposite to our hypothesis, the septic patients had significantly lower plasma TMAO concentrations than did patients admitted for CAG (1.0 vs. 3.0 μmol/L, *p* < 0.001; Additional file 1: Fig. 1). Relative to patients admitted for CAG, septic patients were with poor nutritional status, reflected by lower BMIs and higher NRS-2002 scores (Additional file 1: Table 1), and received more antibiotic treatments before they admitted to ICU.

**Table 1:** Baseline characteristics and nutritional status of septic patients according to plasma TMAO concentration

	Low TMAO (< 0.4 μmol/L) *N* = 32	Median TMAO (0.4–2.5 μmol/L) *N* = 32	High TMAO (≥ 2.5 μmol/L) *N* = 31	*p* value
Age (years)	68.5 (61.3–79.5)	67.5 (56.3–74.8)	70.0 (57.0–78.0)	0.371
Male gender	17 (53.1)	18 (56.3)	23 (74.2)	0.182
Smoking	3 (9.4)	6 (18.2)	10 (32.3)	0.074
Disease severity				
APACHE II scores	29 (28.0–31.0)	28.0 (23.3–31.0)	29.0 (24.0–32.0)	0.742
SOFA scores	12.0 (10.0–12.8)	11.0 (9.0–13.8)	11.0 (9.0–12.0)	0.662
Mean arterial pressure (mmHg)	56.7 (50.3–63.5)	58.3 (50.2–65.0)	53.0 (46.3–61.3)	0.163
Septic shock	14 (43.8)	12 (37.5)	8 (25.8)	0.322
Causes & treatment of sepsis				
Respiratory tract infection	26 (81.3)	24 (75.0)	26 (83.9)	0.663
Urinary tract infection	0 (0.0)	3 (9.4)	3 (9.7)	0.196
Intra-abdominal infection	8 (25.0)	7 (21.9)	3 (9.7)	0.262
Bloodstream infection	17 (53.1)	17 (53.1)	9 (29.0)	0.087
Abx pre-treatment, numbers	5.5 (3.0–7.0)	3.0 (2.3–4.8)	2.0 (1.0–3.0)	< 0.001
Abx pre-treatment, days	12.5 (3.3–27.8)	3.0 (2.0–10.0)	1.0 (1.0–4.0)	< 0.001
Co-morbidities				
Hypertension	9 (28.1)	13 (40.6)	15 (48.4)	0.250
Diabetic mellitus	7 (21.9)	8 (25.0)	8 (25.8)	0.928
Heart failure	4 (12.5)	2 (6.3)	2 (6.5)	0.594
COPD	3 (9.4)	3 (9.4)	1 (3.2)	0.561
Cirrhosis	2 (6.3)	3 (9.4)	0 (0.0)	0.238
Prior CAD	1 (3.1)	2 (6.3)	8 (25.8)	0.010
Prior stroke or PAD	1 (3.1)	1 (3.1)	5 (16.1)	0.075
Malignancy	20 (62.5)	15 (46.9)	14 (45.2)	0.313
Autoimmune disease	2 (6.3)	6 (18.6)	4 (12.9)	0.322
Laboratory data				
White blood cells (K)	9.0 (3.3–15.0)	9.6 (1.1–16.2)	8.6 (2.1–12.4)	0.906
Hemoglobin (mg/dL)	8.6 (7.7–9.3)	8.8 (7.7–10.0)	8.4 (7.6–9.8)	0.722
eGFR (mL/min/1.73m^2^)	57.4 (26.4–90.3)	43.0 (21.8–78.9)	21.5 (7.4–35.2)	0.001
Total bilirubin (mg/dL)	1.8 (0.7–3.1)	1.7 (0.8–4.5)	0.8 (0.4–1.7)	0.008
Glucose (mg/dL)	132.0 (97.8–205.8)	133.0 (101.3–204.0)	147.0 (107.0–228.0)	0.697
Lactate (mg/dL)	18.3 (8.3–25.4)	16.7 (9.9–24.1)	10.8 (7.2–19.8)	0.268
C-reactive protein (mg/dL)	7.5 (3.0–13.4)	12.2 (6.9–19.3)	13.5 (5.9–25.0)	0.030
TMAO (μmol/L)	0.1 (0.1–0.2)	1.1 (0.7–1.9)	4.7 (3.5–11.0)	< 0.001
Nutritional status				
Enteral intake of calories (kcal/day)	89.5 (0.0–623.5)	502.0 (0.0–768.9)	1355.5 (1055.0–1573.0)	< 0.001
Enteral intake of protein (g/day)	3.5 (0.0–25.9)	20.0 (0.0–38.4)	61.0 (35.0–71.0)	< 0.001
Total calories (% of target)	30.9 (12.6–51.8)	43.5 (27.9–68.0)	81.7 (67.1–110.3)	< 0.001
Total protein (% of target)	14.6 (0.0–53.1)	32.0 (0.0–59.0)	66.9 (55.4–86.2)	< 0.001
Body mass index	19.3 (17.7–20.3)	21.3 (18.7–25.2)	23.9 (22.4–28.6)	< 0.001
Albumin (mg/dL)	2.8 (2.6–3.2)	2.9 (2.5–3.2)	3.3 (2.9–3.8)	0.001
Prealbumin (mg/dL)	6.9 (4.4–10.9)	7.7 (5.4–13.1)	12.0 (8.3–17.0)	0.001
Nutrition risk index	80.1 (73.8–85.5)	84.5 (78.5–92.4)	98.3 (87.3–109.8)	< 0.001
NRS 2002 scores	5.0 (5.0–6.0)	4.0 (4.0–5.8)	4.0 (3.0–4.0)	< 0.001

*TMAO* trimethylamine-*N*-oxide; *APACHE* Acute Physiology and Chronic Health Evaluation; *SOFA* Sequential Organ Failure Assessment; *Abx* antibiotic; *COPD* chronic obstructive pulmonary disease; *CAD* coronary artery disease; *PAD* peripheral arterial disease; *eGFR* estimated glomerular filtration rate; *NRS-2002* Nutritional Risk Screening 2002

**Fig. 2:**
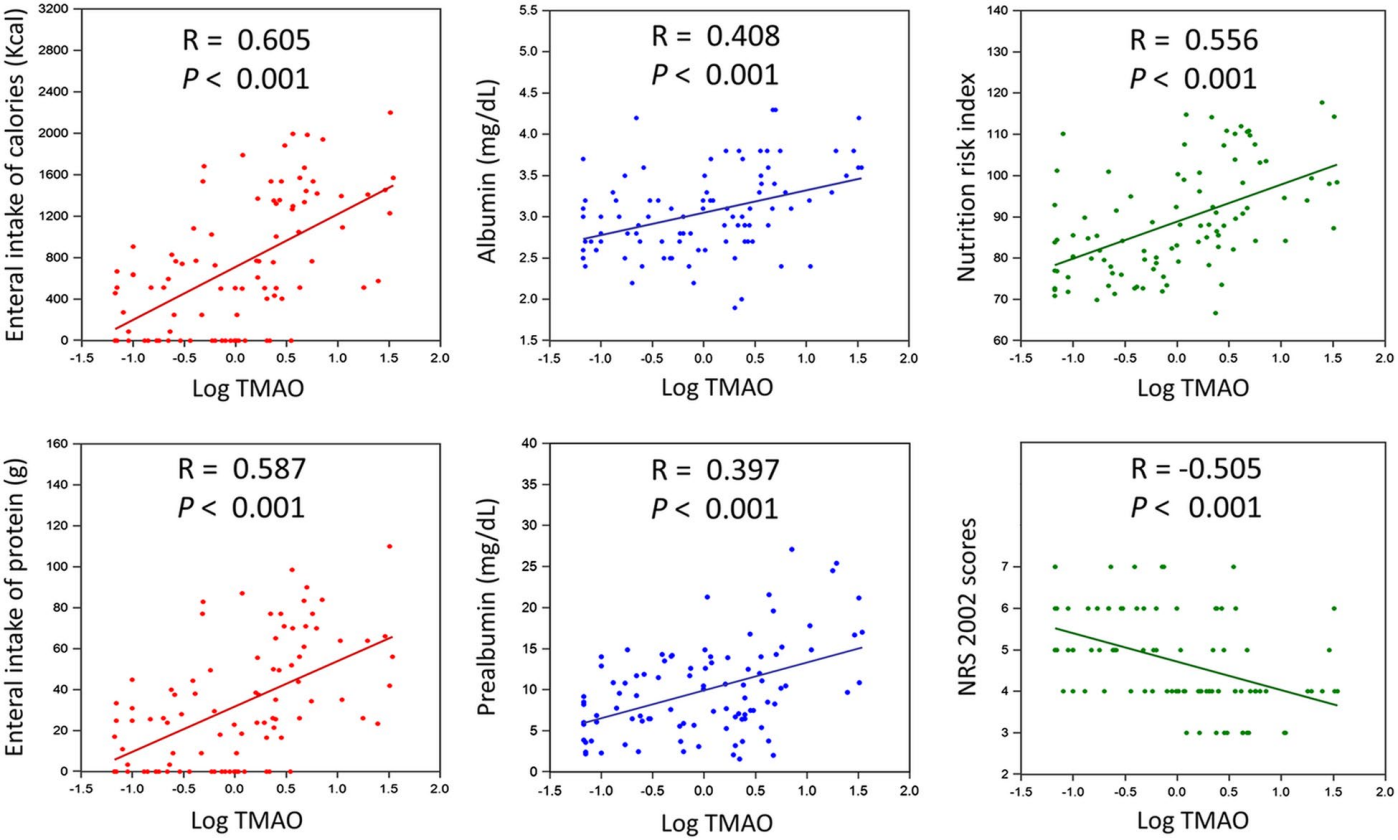
Correlations of (log-transformed) plasma TMAO concentrations with nutritional indicators in septic patients. *TMAO* trimethylamine-*N*-oxide; *NRI* nutrition risk index; *NRS 2002 score* nutrition risk screening 2002 scores

Among septic patients, plasma TMAO levels correlated significantly with enteral calorie (*r* = 0.605) and protein (*r* = 0.587) intakes, albumin (*r* = 0.408) and prealbumin (*r* = 0.397) level, NRI (*r* = 0.556), and NRS 2002 (*r* = − 0.505) scores (all *p* < 0.001; Fig. 2). In the univariate linear regression analyses, TMAO concentrations correlated positively with prior CAD, the CRP level, daily calorie and protein intakes, the BMI, the albumin, prealbumin level, and the NRI; and negatively with the main arterial pressure (MAP), septic shock, number and duration of previous antibiotic treatment courses, eGFR at the time of ICU admission, and NRS-2002 score (showed in Table 2). In the multivariate regression model, the MAP, septic shock, number of previous antibiotic treatment courses, eGFR, enteral calorie intake, prealbumin, and NRI remained significantly correlated with the TMAO concentration.

**Table 2:** Univariate and multivariate linear regression analyses of factors associated with plasma TMAO concentration (log transformation to achieve normality before analysis) in septic patients

	Univariate analysis		Multivariate analysis*	
	Std *β*	*p* value	Std *β*	*p* value
Age	0.018	0.866		
Male gender	0.153	0.139		
Smoker	0.145	0.161		
APACHE II scores	− 0.090	0.387		
SOFA scores	− 0.045	0.663		
Mean arterial pressure	− 0.248	0.015	− 0.215	0.001
Septic shock	− 0.202	0.050	− 0.155	0.019
Respiratory tract infection	− 0.007	0.943		
Urinary tract infection	0.124	0.231		
Intra-abdominal infection	− 0.161	0.119		
Bloodstream infection	− 0.131	0.205		
Antibiotic pre-treatment, numbers	− 0.644	< 0.001	− 0.337	< 0.001
Antibiotic pre-treatment, days	− 0.375	< 0.001		
Hypertension	0.170	0.100		
Diabetic mellitus	0.088	0.398		
Heart failure	− 0.030	0.773		
COPD	− 0.114	0.270		
Cirrhosis	− 0.041	0.691		
Prior CAD	0.294	0.004		
Prior stroke or PAD	0.145	0.162		
Malignancy	− 0.110	0.291		
Autoimmune disease	0.113	0.276		
White blood cells (K)	0.047	0.653		
Hemoglobin	0.011	0.916		
eGFR at ICU admission	− 0.357	< 0.001	− 0.156	0.015
Total bilirubin	− 0.161	0.120		
Glucose	0.082	0.427		
Lactate	− 0.059	0.573		
C-reactive protein	0.261	0.011		
Enteral intake of calories	0.602	< 0.001	0.279	< 0.001
Enteral intake of protein	0.566	< 0.001		
Total calories (% of target)	0.573	< 0.001		
Total protein (% of target)	0.500	< 0.001		
Body mass index	0.380	< 0.001		
Albumin	0.397	< 0.001		
Prealbumin	0.456	< 0.001	0.157	0.025
Nutrition risk index	0.519	< 0.001	0.187	0.007
NRS 2002 scores	− 0.453	< 0.001		

*TMAO* trimethylamine-*N*-oxide; *APACHE* Acute Physiology and Chronic Health Evaluation; *SOFA* Sequential Organ Failure Assessment; *CAD* coronary artery disease; *CI* confidence interval; *COPD* chronic obstructive pulmonary disease; *PAD* peripheral arterial disease; *eGFR* estimated glomerular filtration rate; *HR* hazard ratio; *NRS-2002* Nutritional Risk Screening 2002

*Adjusted for variables with *p* < 0.1 in the univariate analysis

### Outcomes of septic patients according to TMAO concentration

**Fig. 3:**
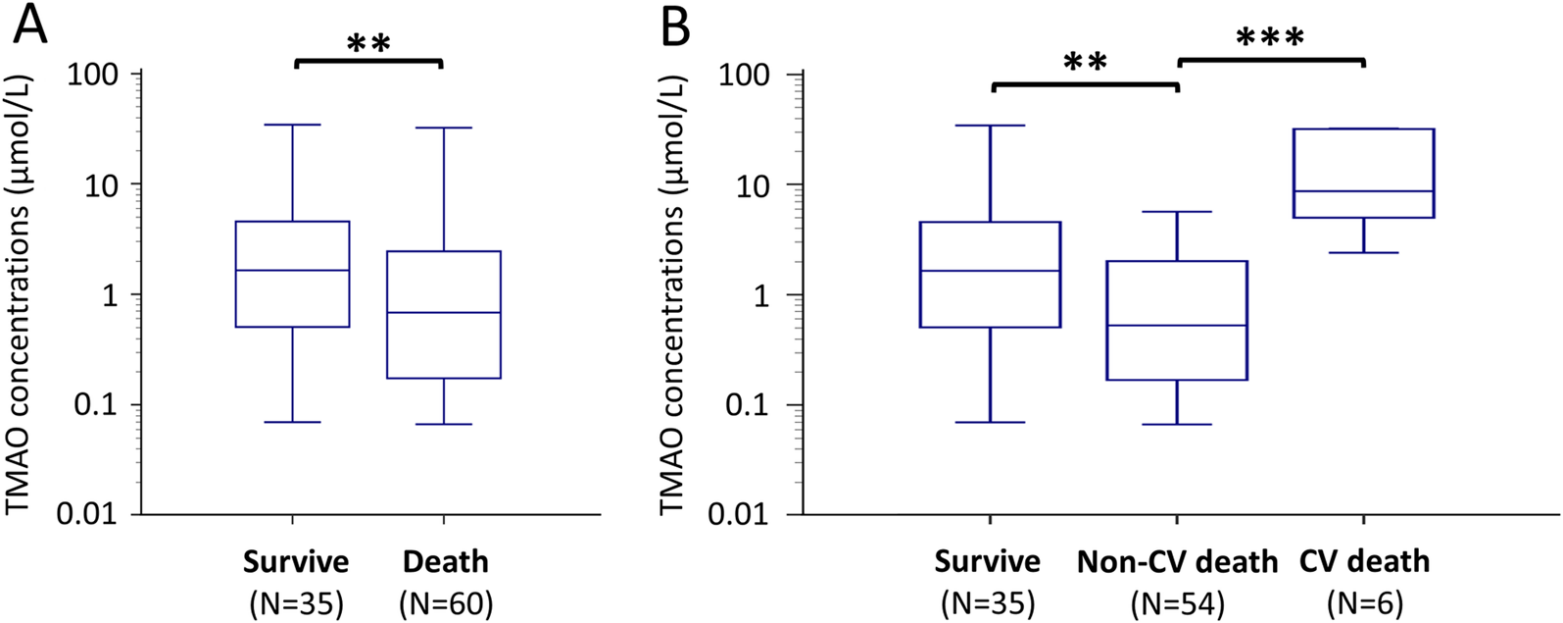
Plasma TMAO concentrations in patients classified by **A** in-hospital death or survival to discharge and **B** cause of death during hospitalization. **p < 0.05, ***p < 0.001. *TMAO* trimethylamine-*N*-oxide; *CAG* coronary angiography; *CV* cardiovascular

Totally 60 (63.1%) cases of all-cause death (6 CV and 54 non-CV death) occurred during hospitalization among septic patients admitted to the ICU. Compared to survivors, patients who died in hospital had lower TMAO concentrations (*p* = 0.0034; Fig. 3A). These concentrations were also lower among patients who died of non-CV cause than among those who died of CV cause (*p* < 0.001; Fig. 3B). Compared to survivors, patients died of non-CV cause were with significantly lower enteral intake of calories, total proteins, and lower prealbumin levels (Additional file 1: Table 2). In contrast, patients died of CV cause were with better nutritional conditions.

**Fig. 4:**
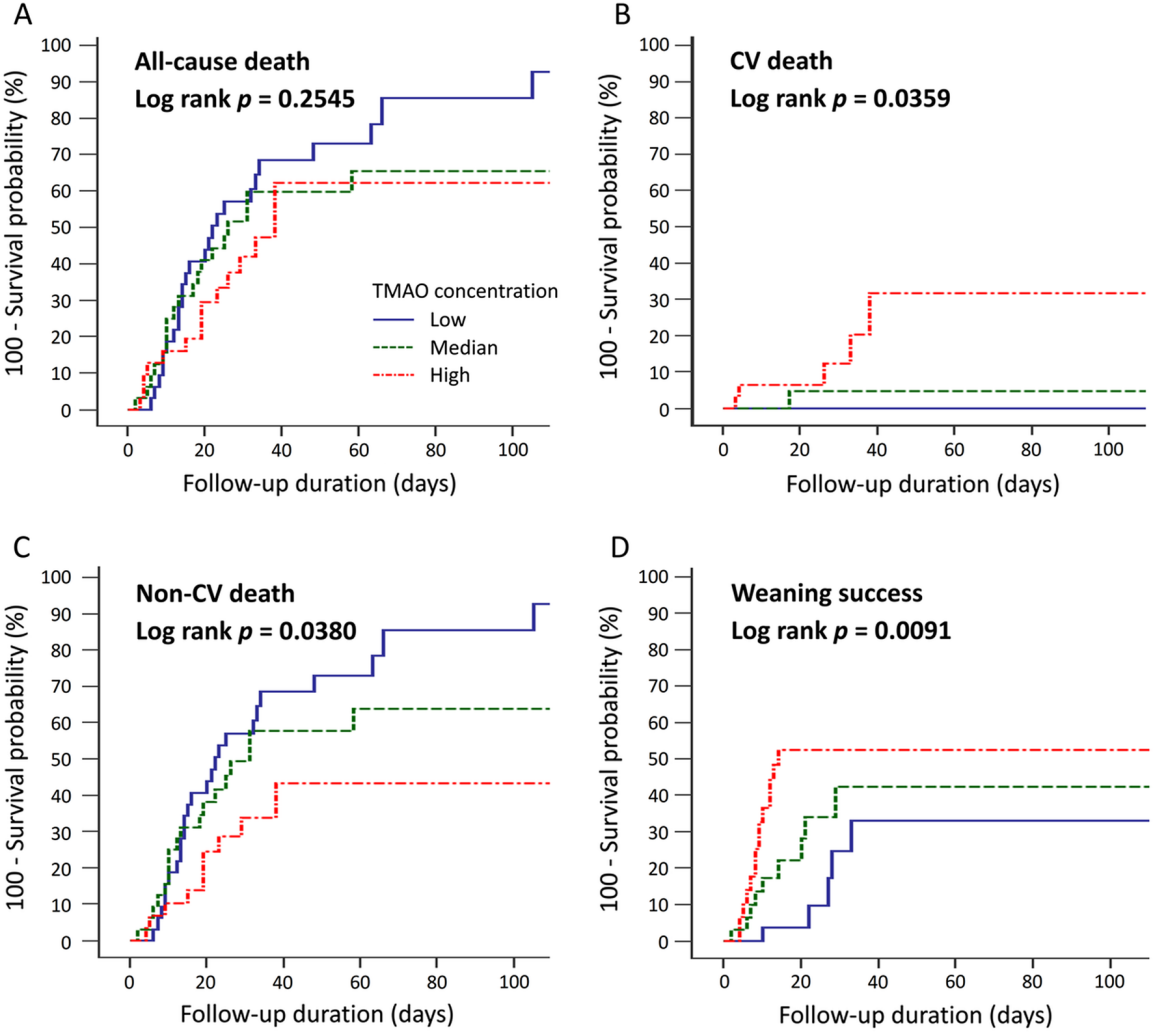
Kaplan–Meier curves for the cumulative incidences of **A** all-cause death, **B** CV death, **C** non-CV death, and **D** ventilator weaning success in septic patients according to the plasma TMAO concentration. *CV* cardiovascular; *TMAO* trimethylamine-*N*-oxide

Clinical outcomes of patients grouped by TMAO tertiles are summarized in Table 3. The incidence of all-cause death did not differ significantly among the three groups. However, the incidence of CV death was significantly higher among patients with high TMAO concentrations (16.1%) than among those with low and medium concentrations (0% and 3.1%, respectively; *p* = 0.021). Patients with higher TMAO levels also tended to have a higher incidence of AKI within 48 h after ICU admission, although this difference was not significant. In contrast, patients with low plasma TMAO concentrations had a significantly higher incidence of non-CV death [78.1% vs. 59.4% (medium) and 32.3% (high); *p* = 0.001]. Patients with low TMAO concentrations also had a lower rate of successful ventilator weaning [15.6% vs. 28.1% (medium) and 45.2% (high); *p* = 0.036] and longer duration of ventilator use [21.5 vs. 15.5 (medium) and 12.0 (high) days; *p* = 0.039]. The median lengths of ICU and hospital stays among all septic patients were 11.0 and 23.0 days, respectively, and did not differ according to TMAO concentration. The cumulative incidences of death and successful ventilator weaning, stratified by TMAO concentrations, are shown in Fig. 4. Low TMAO concentrations were associated with a lower incidence of CV death (log-rank *p* = 0.0359), higher incidence of non-CV death (log-rank *p* = 0.0380), and lower rate of successful ventilator weaning (log-rank *p* = 0.0091).

**Table 3:** Clinical outcomes of septic patients according to plasma TMAO concentration

	Low TMAO (< 0.4 μmol/L) *N* = 32	Median TMAO (0.4–2.5 μmol/L) *N* = 32	High TMAO (≥ 2.5 μmol/L) *N* = 31	*p* value
Primary outcomes				
In-hospital, all-cause death	25 (78.1)	20 (62.5)	15 (48.4)	0.050
Cardiovascular death*	0 (0)	1 (3.1)	5 (16.1)	0.021
Non-cardiovascular death^†^	25 (78.1)	19 (59.4)	10 (32.3)	0.001
Secondary outcomes				
Acute kidney injury (AKI)	9 (28.1)	13 (40.6)	18 (58.1)	0.054
AKI required dialysis	3 (9.4)	7 (21.9)	9 (29.0)	0.142
Weaning success	5 (15.6)	9 (28.1)	14 (45.2)	0.036
Length of ventilator usage, days	21.5 (12.3–33.0)	15.5 (8.3–28.3)	12.0 (6.0–23.0)	0.039
Length of ICU stay, days	13.0 (8.3–20.0)	10.5 (6.3–19.5)	10.0 (7.0–16.0)	0.332
Length of hospitalization, days	21.5 (13.0–34.8)	23.5 (10.5–50.0)	26.0 (19.0–37.0)	0.906

*TMAO* trimethylamine-*N*-oxide; *ICU* intensive care unit

*Patients died of myocardial infarction, sudden cardiac death, heart failure, stroke, or CV procedures

^†^Patients died of other causes, primarily sepsis and terminal cancer

### Independent predictors of non-CV death and successful ventilator weaning

Compared with patients with high TMAO levels, patients with low plasma TMAO concentrations were at significantly greater risk of non-CV death, according to the univariate Cox regression analysis [hazard ratio (HR) 2.51, 95% confidence interval (CI) 1.21–5.24, *p* = 0.014]. However, TMAO was no more associated with non-CV death in the multivariate model adjusting septic shock, total bilirubin, enteral intake of calories, enteral intake of protein, total calories, and total protein (Additional file 1: Table 3). In fact, the significant association between TMAO and non-CV death remained even after adjusting the APACHE II and SOFA scores (adjusted HR 2.48, 95% CI 1.10–5.61, *p* = 0.029, in Additional file 1: Table 4). However, the strength of the association diminished after further adjusting the NRS-2002 score (adjusted HR 1.99, 95% CI 0.86–4.50, *p* = 0.108). On the other hand, NRS-2002 score was significantly associated with non-CV death in the multivariate regression analysis (adjusted HR 1.35, 95% CI 1.01–1.81, *p* = 0.046). Plasma TMAO concentration was not an independent predictor for non-CV death in the multivariate regression model. Independent predictors for non-CV death were the APACHE II and SOFA scores, respiratory tract infection, malignancy, lactate concentration, and NRS-2002 score (Table 4).

**Table 4:** Factors associated with non-cardiovascular death among septic patients in univariate and multivariate analyses

	Univariate		Multivariate*	
	Crude HR (95% CI)	*p*	Adjusted HR (95% CI)	*p*
Plasma TMAO				
High (≥ 2.5 μmol/L)	Ref	Ref		
Median (0.4–2.5 μmol/L)	1.80 (0.83–3.91)	0.137		
Low (< 0.4 μmol/L)	2.51 (1.21–5.24)	0.014		
Age	1.00 (0.98–1.02)	0.753		
Male gender	1.61 (0.90–2.88)	0.112		
Smoker	1.11 (0.57–2.16)	0.765		
APACHE II scores	1.11 (1.05–1.16)	< 0.001	1.11 (1.04–1.18)	0.001
SOFA scores	1.22 (1.12–1.32)	< 0.001	1.18 (1.06–1.31)	0.002
Mean arterial pressure	0.99 (0.97–1.01)	0.374		
Septic shock	1.74 (0.99–3.06)	0.053		
Respiratory tract infection	0.50 (0.26–0.94)	0.031	0.31 (0.16–0.62)	0.001
Urinary tract infection	0.50 (0.12–2.05)	0.333		
Intra-abdominal infection	1.59 (0.83–3.04)	0.164		
Bloodstream infection	0.91 (0.53–1.57)	0.731		
Antibiotic pre-treatment, numbers	1.07 (0.95–1.20)	0.271		
Antibiotic pre-treatment, days	1.01 (0.99–1.02)	0.342		
Hypertension	0.67 (0.38–1.20)	0.180		
Diabetic mellitus	1.06 (0.56–1.98)	0.868		
Heart failure	0.69 (0.21–2.22)	0.532		
COPD	1.19 (0.43–3.32)	0.738		
Cirrhosis	1.23 (0.38–3.95)	0.731		
Prior CAD	1.00 (0.36–2.80)	0.998		
Prior stroke or PAD	0.33 (0.08–1.38)	0.129		
Malignancy	1.82 (1.04–3.20)	0.037	2.08 (1.14–3.79)	0.018
Autoimmune disease	0.39 (0.12–1.25)	0.114		
White blood cells (K)	0.99 (0.96–1.03)	0.743		
Hemoglobin	0.97 (0.84–1.12)	0.667		
eGFR at ICU admission	1.00 (1.00–1.01)	0.457		
Total bilirubin	1.06 (1.01–1.11)	0.014		
Glucose	1.00 (1.00–1.00)	0.945		
Lactate	1.02 (1.01–1.03)	0.003	1.02 (1.01–1.03)	0.002
C-reactive protein	0.98 (0.95–1.01)	0.231		
Enteral intake of calories	1.00 (1.00–1.00)	0.064		
Enteral intake of protein	0.99 (0.98–1.00)	0.087		
Total calories (% of target)	0.99 (0.99–1.00)	0.088		
Total protein (% of target)	0.99 (0.99–1.00)	0.099		
Body mass index	0.97 (0.92–1.03)	0.359		
Albumin	0.78 (0.47–1.31)	0.354		
Prealbumin	0.92 (0.87–0.97)	0.002		
Nutrition risk index	0.98 (0.96–1.01)	0.156		
NRS 2002 scores	1.26 (0.99–1.60)	0.057	1.43 (1.10–1.86)	0.008

*HR* hazard ratio; *CI* confidence interval; *TMAO* trimethylamine-*N*-oxide; *Ref* reference; *APACHE* Acute Physiology and Chronic Health Evaluation; *SOFA* Sequential Organ Failure Assessment; *COPD* chronic obstructive pulmonary disease; *CAD* coronary artery disease; *PAD* peripheral arterial disease; *eGFR* estimated glomerular filtration rate; *NRS-2002* Nutritional Risk Screening 2002

*Adjusted for variables with *p* < 0.1 in the univariate analysis

Compared with patients with high TMAO levels, patients with low plasma TMAO concentrations had a significantly lower successful ventilator weaning rate (HR 0.23, 95% CI 0.08–0.65, *p* = 0.005). The plasma TMAO concentration remained an independent predictor of successful ventilator weaning after adjustment for the APACHE II score and CRP concentration in the multivariate regression model (adjusted HR 0.32, 95% CI 0.11–0.91, *p* = 0.033; showed in Additional file 1: Table 5).

## Discussion

This prospective study investigated the relationships of TMAO concentrations and mortality in septic patients. Septic patients had significantly lower plasma TMAO concentrations than did subjects admitted for CAG. Compared with those in the highest TMAO tertile, septic patients in the lowest TMAO tertile were at greater risk of in-hospital death and unsuccessful ventilator weaning, which may be characterized as the TMAO paradox. However, the strength of the association diminished after adjustment for the disease severity and NRS-2002 score. The inverse association between plasma TMAO and non-CV death was confounded by the nutritional status. Patients in the lowest TMAO tertile had poorer nutrition status and were given longer antibiotic treatment courses before ICU admission. TMAO concentrations correlated positively with the daily energy intake, albumin and prealbumin concentrations. These findings indicate that TMAO, a microbiota-derived metabolite, may be a novel risk biomarker and a nutritional indicator for septic patients; and provide new insight into the impact of malnutrition in the septic population.

TMAO has been reported to stimulate intracellular reactive oxygen species production and release inflammatory cytokines [8]. Although some in vitro findings suggest that TMAO has protective effects, such as protein [33] and nucleic acid [34] stabilization, elevated TMAO concentrations are generally considered to be harmful and have been linked to various CV diseases [2–5, 9]. Contrary to our hypothesis, septic patients in the lowest TMAO tertile were at greater risk of non-CV death and unsuccessful ventilator weaning than were those in higher tertiles. Three rationales may explain the inverse association between the TMAO concentration and adverse outcomes. The first explanation is the impact of malnutrition. As TMAO originates from the bacterial metabolism of dietary choline or carnitine [7], its concentration depends largely on enteral nutrition. Septic patients in the lowest TMAO tertile had lower BMIs and higher NRS-2002 scores, which suggest premorbid malnutrition and thus increased vulnerability to acute stress. Malnutrition was also reported to be associated with intestinal dysbiosis and metabolic endotoxemia [35], which may further deteriorate sepsis. Another explanation is the influence of antibiotic treatment before ICU admission. In a previous study, plasma TMAO concentrations became undetectable after 1 week of broad-spectrum antibiotic treatment [9]. The median duration of pre-ICU admission antibiotic treatment among septic patients in our study was 3 days. This treatment thus considerably reduced TMAO concentrations. The third explanation involves the influence of organ dysfunction during sepsis. Sepsis may result in intestinal dysfunction and dysbiosis [36], which directly reduced the enteral intake and attenuated the production of bacterial metabolites. Moreover, trimethylamine, the precursor of TMAO, was converted into TMAO by liver enzymes (flavin-containing monooxygenase-3) [7]. Sepsis-associated liver dysfunction may also suppress the conversion and decrease TMAO concentration. Finally, TMAO is eliminated by kidneys [12]. Both sepsis-associated AKI and dialysis may affect its plasma concentration [37]. Theses explanations were supported by the results of our analyses. Enteral intake of calories, the numbers of previous antibiotic treatment courses, eGFR, and NRI (composed of albumin and BMI) were all independently correlated with plasma TMAO concentration in the multivariate regression analysis. However, only the nutritional indicator, NRS-2002 score, was independently associated with non-CV death. For septic patients, the impact of malnutrition was somewhat greater than those of inflammation or previous antibiotic treatment.

Several pieces of clinical evidence support the association between TMAO and nutrition. The dietary content may modulate the production of TMAO; plasma TMAO concentrations have been found to increase with high-protein [38] and high-fat [39] diets, and to decrease upon supplementation with indigestible carbohydrates [40]. Circulating TMAO levels have been found to correlate positively with energy intake [41], the consumption of animal proteins [41], the BMI [4, 41], and the albumin concentration [42]. Compatible with these previous observations, plasma TMAO concentrations correlated positively with septic patients’ daily calorie and protein intakes, BMIs, and albumin concentrations in this study. Not surprisingly, they also correlated with other nutritional indicators, including the NRI and NRS-2002 score. These findings suggest that septic patients with low plasma TMAO levels have both acute and chronic disease-related malnutrition. As a potential nutritional indicator, TMAO has obvious weak point. The measurement of plasma TMAO is expensive and easy to be influenced by the antibiotic therapy. Nevertheless, TMAO has the strengths to reflect the absorption of animal proteins (including fishes, eggs, and meats) [43] and gut microbiota metabolism [44], which cannot not be substituted by other biomarkers.

TMAO may play different roles in CV and infectious diseases. Choline-rich diets and TMAO are frequently reported to be risk factors for CV diseases [2, 3, 9]. In contrast, we observed an inverse association between the circulating TMAO level and non-CV death among septic patients in this study. One observational study suggested that plasma TMAO levels correlated positively with long-term mortality in a population of patients with community-acquired pneumonia without CAD [31]. However, those patients had much less-severe disease and higher BMIs and plasma TMAO values at the time of enrollment (median, 3.0 μmol/L) than did our septic patients. In another study, circulating TMAO concentrations were lower in untreated patients with human immunodeficiency virus infection and increased significantly after treatment initiation (from 1.28 to 2.30 μmol/L), eventually becoming similar to those of healthy subjects [45]. The inverse association between the TMAO concentration and non-CV death in our study may be seen only in the advanced stage of sepsis or in extremely ill patients. Changes in the plasma TMAO concentration at different stages of sepsis should be examined in further longitudinal studies.

Several limitations of this study should be addressed. First, the study was conducted at a single center with small patient groups. The clinical significance of the TMAO concentration for all-cause mortality should be investigated in a large-scale study. In addition, the septic patients enrolled in this study were geriatric and had relatively severe disease, which limits the generalizability of our results. Second, in the absence of indirect calorimetry, we used weight-based equations to determine the energy requirements, which may be less accurate in critically ill patients. On top of that, we only used the BMI, NRI, and NRS-2002 score, which routinely calculated in our hospital, to screen for nutrition risk among the septic patients. Other nutritional indicators, such as muscle mass, body fat, and other body composition measurements, are not included in the diagnostic criteria for malnutrition [46]. Third, we did not obtain stool samples to analyze the microbiome of enrolled patients. Septic and critically ill patients were reported to have decreased intestinal microbiota diversity [6], and gut dysbiosis was also linked to reduced TMAO levels in previous observation [47]. An additional study with bioinformatic analysis should be performed to confirm the association between dysbiosis and plasma TMAO in septic patients.

## Conclusions

The circulating TMAO level is associated inversely with non-CV death among extremely ill septic patients, as the TMAO paradox. For these patients, the impact of malnutrition is somewhat greater than the pro-inflammatory effect of TMAO. This study provides indirect evidence for the impact of malnutrition on intestinal microbiota. Further studies are need to investigate the relationship between intestinal dysfunction, nutritional status, and gut microbiota in the septic population.

## Supplementary Information

Additional file 1: **Figure 1.** Plasma TMAO concentrations in patients classified by **A** reason for hospitalization (sepsis vs. elective CAG) and **B** sepsis and different severity of CAD. **Table 1.** Baseline characteristics, TMAO concentrations, and outcomes of septic patients and patients admitted for elective CAG. **Table 2.** Plasma TMAO concentrations and nutritional indicators of patients classified by different causes of death during hospitalization. **Table 3.** Univariate and multivariate Cox regression analyses to investigate the relationships between TMAO, septic shock, total bilirubin, enteral intake of calories, enteral intake of protein, total calories, total protein, and non-cardiovascular death among septic patients. **Table 4.** Univariate and multivariate Cox regression analyses to investigate the relationships between TMAO, disease severity, antibiotic pre-treatment, nutritional risk scores, and non-CV death among septic patients. **Table 5.** Univariate and multivariate Cox regression analyses of factors associated with weaning success among septic patients

## Data Availability

The data sets generated during and analyzed during the current study are available from the corresponding author on reasonable request.

## Abbreviations

AKI: Acute kidney injury
AMI: Acute myocardial infarction
APACHE II: Acute Physiology and Chronic Health Evaluation II
BMI: Body mass index
CAD: Coronary artery disease
CAG: Coronary angiography
CI: Confidence interval
CRP: C-reactive protein
CV: Cardiovascular
HR: Hazard ratio
MAP: Mean arterial pressure
NRI: Nutrition risk index
NRS-2002: Nutrition Risk Screening 2002
SOFA: Sequential Organ Failure Assessment
TMAO: Trimethylamine *N*-oxide
