# Supplementary material for: Paradox of trimethylamine-N-oxide, the impact of malnutrition on microbiota-derived metabolites and septic patients
Source: J Intensive Care. 2021 Oct 21;9:65. doi: 10.1186/s40560-021-00581-5 (PMC8529374; doi:10.1186/s40560-021-00581-5)
Supplement: Supplementary file 1 — Additional file 1: Figure 1. Plasma TMAO concentrations in patients classified by A reason for hospitalization (sepsis vs. elective CAG) and B sepsis and different severity of CAD. Table 1. Baseline characteristics, TMAO concentrations, and outcomes of septic patients and patients admitted for elective CAG. Table 2. Plasma TMAO concentrations and nutritional indicators of patients classified by different causes of death during hospitalization. Table 3. Univariate and multivariate Cox regression analyses to investigate the relationships between TMAO, septic shock, total bilirubin, enteral intake of calories, enteral intake of protein, total calories, total protein, and non-cardiovascular death among septic patients. Table 4. Univariate and multivariate Cox regression analyses to investigate the relationships between TMAO, disease severity, antibiotic pre-treatment, nutritional risk scores, and non-CV death among septic patients. Table 5. Univariate and multivariate Cox regression analyses of factors associated with weaning success among septic patients [file 40560_2021_581_MOESM1_ESM.docx]

**Paradox of trimethylamine-N-oxide, the impact of malnutrition on microbiota-derived metabolites and septic patients**

Ruey-Hsing Chou^1,2,3,4^, Po-Shan Wu^5^, Shen-Chih Wang^6^, Cheng-Hsueh Wu^1,2^, Shu-Fen Lu^7,8^, Ru-Yu Lien^7,8^, Yi-Lin Tsai^1,3^, Ya-Wen Lu^1,3^, Ming-Ren Kuo^1,3^, Jiun-Yu Guo^1,3^, Ruey-Yi Chou^9^, Po-Hsun Huang^1,2,3,4,^*, Shing-Jong Lin^3,4,10,11,12^

^1^ Division of Cardiology, Department of Medicine, Taipei Veterans General Hospital, Taipei, Taiwan;

^2^ Department of Critical Care Medicine, Taipei Veterans General Hospital, Taipei, Taiwan;

^3^ Cardiovascular Research Center, National Yang Ming Chiao Tung University, Taipei, Taiwan;

^4^ Institute of Clinical Medicine, National Yang Ming Chiao Tung University, Taipei, Taiwan;

^5^ Division of Clinical Nutrition, Department of Dietetics and Nutrition, Taipei Veterans General Hospital, Taipei, Taiwan;

^6^ Department of Anesthesiology, Taipei Veterans General Hospital, Taipei, Taiwan;

^7^ Department of Nursing, Taipei Veterans General Hospital, Taipei, Taiwan;

^8^ School of Nursing, National Yang Ming Chiao Tung University, Taipei, Taiwan;

^9^Division of Gastroenterology and Hepatology, Department of Medicine, Taipei Veterans

General Hospital, Taipei, Taiwan

^10^ Healthcare and Services Center, Taipei Veterans General Hospital, Taipei, Taiwan;

^11^ Taipei Heart Institute, Taipei Medical University, Taipei, Taiwan;

^12^ Division of Cardiology, Heart Center, Cheng-Hsin General Hospital, Taipei, Taiwan.

**Additional figure 1.** Plasma TMAO concentrations in patients classified by **(A)** reason for hospitalization (sepsis vs. elective CAG) and **(B)** sepsis and different severity of CAD.


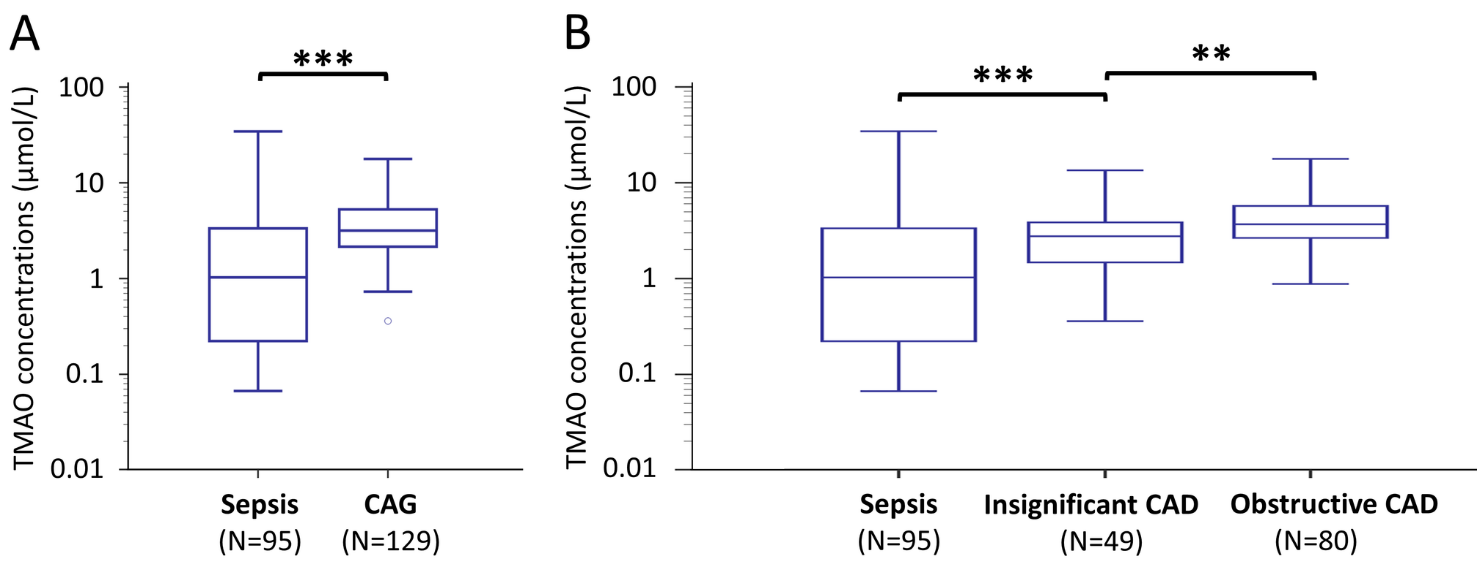


** *P* < 0.05, ****P* < 0.001

TMAO, trimethylamine-N-oxide; CAG, coronary angiography; CV, cardiovascular.

**Additional table 1.** Baseline characteristics, TMAO concentrations, and outcomes of septic patients and patients admitted for elective CAG.

|  | **CAG group**  N=129 | **Sepsis group**  N=95 | ***P***  **value** |
| --- | --- | --- | --- |
| Age (years) | 71.0 (60.5-77.0) | 70.0 (60.0-78.0) | 0.899 |
| Male gender | 86 (66.7) | 58 (61.1) | 0.401 |
| Smoking | 50 (38.8) | 19 (20.0) | 0.003 |
| Mean arterial pressure (mmHg) | 102.0 (92.5-108.2) | 55.3 (50.0-63.7) | <0.001 |
| Abx pre-treatment, numbers | 0 (0.0) | 3.0 (2.0-5.0) | <0.001 |
| Abx pre-treatment, days | 0 (0.0) | 3.0 (1.0-13.0) | <0.001 |
| **Co-morbidities** |  |  |  |
| Hypertension | 79 (61.2) | 37 (38.9) | 0.001 |
| Diabetic mellitus | 44 (34.1) | 23 (24.2) | 0.140 |
| Heart failure | 8 (6.2) | 8 (8.4) | 0.603 |
| Prior CAD | 7 (5.4) | 11 (11.6) | 0.134 |
| Prior stroke or PAD | 12 (9.3) | 7 (7.4) | 0.809 |
| **Laboratory data** |  |  |  |
| White blood cells (K) | 6.7 (5.9-8.0) | 8.7 (2.8-14.5) | 0.022 |
| Hemoglobin (mg/dL) | 13.1 (11.6-14.2) | 8.6 (7.6-9.8) | <0.001 |
| eGFR (mL/min /1.73m^2^) | 69.8 (59.0-81.0) | 32.9 (16.7-80.4) | <0.001 |
| Glucose (mg/dL) | 99.0 (91.0-123.5) | 135.0 (102.0-206.0) | <0.001 |
| C-reactive protein (mg/dL) | 0.1 (0.0-0.4) | 9.7 (5.7-19.2) | <0.001 |
| TMAO (μmol/L) | 3.2 (2.1-5.3) | 1.0 (0.2-3.5) | <0.001 |
| **Nutritional** **status** |  |  |  |
| Body mass index | 25.5 (23.5-27.8) | 21.5 (18.7-25.2) | <0.001 |
| NRS 2002 scores | 1.0 (0.0-1.0) | 4.0 (4.0-6.0) | <0.001 |

TMAO, trimethylamine-N-oxide; CAG, coronary angiography; Abx, antibiotic; CAD, coronary artery disease; PAD, peripheral arterial disease; eGFR, estimated glomerular filtration rate; NRS-2002, Nutritional Risk Screening 2002.

**Additional table 2.** Plasma TMAO concentrations and nutritional indicators of patients classified by different causes of death during hospitalization.

|  | **Survive**  N=35 | **Non-CV death**  N=54 | **CV death**  N=6 | ***P* value** |
| --- | --- | --- | --- | --- |
| TMAO (μmol/L) | 1.7 (0.5-4.7) | 0.5 (0.2-2.1) | 8.9 (4.4-32.1) | <0.001^abc^ |
| Enteral intake of calories (kcal/day) | 754.0 (251.0-1369.5) | 503.5 (0.0-784.1) | 1669.0 (1030.0-2038.5) | 0.004^abc^ |
| Enteral intake of protein (g/day) | 31.0 (9.0-56.0) | 20.8 (0.0-37.8) | 74.0 (36.9-95.0) | 0.006^bc^ |
| Total calories (% of target) | 57.3 (37.0-81.7) | 42.2 (16.2-73.9) | 112.4 (86.9-161.9) | 0.002^bc^ |
| Total protein (% of target) | 51.8 (26.6-69.5) | 29.6 (0.0-58.3) | 75.1 (55.2-123.4) | 0.004^abc^ |
| Body mass index | 23.2 (20.8-25.0) | 19.5 (18.6-25.3) | 24.0 (16.9-27.9) | 0.110 |
| Albumin (mg/dL) | 3.2 (2.6-3.5) | 2.9 (2.7-3.2) | 3.7 (3.2-4.2) | 0.009^bc^ |
| Prealbumin (mg/dL) | 11.7 (6.4-14.9) | 7.5 (5.7-10.3) | 16.1 (10.8-22.7) | <0.001^ac^ |
| ^a^ significant difference (*p* <0.05) between survivors and patients with non-CV death  ^b^ significant difference (*p* <0.05) between survivors and patients with CV death  ^c^ significant difference (*p* <0.05) between patients with non-CV death and with CV death | | | | |

TMAO, trimethylamine-N-oxide; CV, cardiovascular; NRS-2002, Nutritional Risk Screening 2002.

**Additional table 3.** Univariate and multivariate Cox regression analyses to investigate the relationships between TMAO, septic shock, total bilirubin, enteral intake of calories, enteral intake of protein, total calories, total protein, and non-cardiovascular death among septic patients.

|  | **Univariate Multivariate*** | | | | |
| --- | --- | --- | --- | --- | --- |
|  | **Crude HR (95% CI)** | ***P*** |  | **Adjusted HR (95% CI)** | ***P*** |
| Plasma TMAO |  |  |  |  |  |
| High (≥2.5 μmol/L) | Ref | Ref |  | Ref | Ref |
| Median (0.4-2.5 μmol/L) | 1.80 (0.83-3.91) | 0.137 |  | 1.45 (0.61-3.48) | 0.404 |
| Low (<0.4 μmol/L) | 2.51 (1.21-5.24) | 0.014 |  | 1.76 (0.68-4.58) | 0.244 |
| Septic shock | 1.74 (0.99-3.06) | 0.053 |  | 1.67 (0.94-2.97) | 0.081 |
| Total bilirubin | 1.06 (1.01-1.11) | 0.014 |  | 1.06 (1.01-1.12) | 0.027 |
| Enteral intake of calories | 1.00 (1.00-1.00) | 0.064 |  | 1.00 (1.00-1.00) | 0.802 |
| Enteral intake of protein | 0.99 (0.98-1.00) | 0.087 |  | 1.01 (0.94-1.09) | 0.817 |
| Total calories (% of target) | 0.99 (0.99-1.00) | 0.088 |  | 1.00 (0.98-1.01) | 0.550 |
| Total protein (% of target) | 0.99 (0.99-1.00) | 0.099 |  | 1.00 (0.98-1.02) | 0.986 |

*Adjusted for TMAO and variables with *p* < 0.1 in the univariate analysis.

TMAO, trimethylamine-N-oxide; HR, hazard ratio; CI, confidence interval; Ref, reference.

**Additional table 4.** Univariate and multivariate Cox regression analyses to investigate the relationships between TMAO, disease severity, antibiotic pre-treatment, nutritional risk scores, and non-CV death among septic patients.

|  | **Univariate** | | **Multivariate (Model 1)*** | | **Multivariate (Model 2)**† | |
| --- | --- | --- | --- | --- | --- | --- |
|  | **Crude HR (95% CI)** | ***P*** | **aHR (95% CI)** | ***P*** | **aHR (95% CI)** | ***P*** |
| Plasma TMAO |  |  |  |  |  |  |
| High (≥2.5 μmol/L) | Ref | Ref | Ref | Ref | Ref | Ref |
| Median (0.4-2.5 μmol/L) | 1.80 (0.83-3.91) | 0.137 | 2.34 (1.07-5.10) | 0.032 | 1.50 (0.62-3.62) | 0.373 |
| Low (<0.4 μmol/L) | 2.51 (1.21-5.24) | 0.014 | 2.48 (1.10-5.61) | 0.029 | 1.99 (0.86-4.50) | 0.108 |
| APACHE II scores | 1.11 (1.05-1.16) | <0.001 | 1.07 (1.01-1.13) | 0.017 | 1.08 (1.02-1.15) | 0.006 |
| SOFA scores | 1.22 (1.12-1.32) | <0.001 | 1.22 (1.10-1.35) | <0.001 | 1.21 (1.09-1.34) | <0.001 |
| Abx pre-treatment, numbers | 1.07 (0.95-1.20) | 0.271 |  |  |  |  |
| Abx pre-treatment, days | 1.01 (0.99-1.02) | 0.342 |  |  |  |  |
| Nutrition risk index | 0.98 (0.96-1.01) | 0.156 |  |  |  |  |
| NRS 2002 scores | 1.26 (0.99-1.60) | 0.057 |  |  | 1.35 (1.01-1.81) | 0.046 |

*Model 1 (Adjusted for TMAO, APACHE II, SOFA scores)

†Model 2 (Adjusted for TMAO, APACHE II, SOFA scores, and variables with *p* < 0.1 in the univariate analysis)

TMAO, trimethylamine-N-oxide; CV, cardiovascular; APACHE, Acute Physiology and Chronic Health Evaluation; SOFA, Sequential Organ Failure Assessment; NRS- 2002, Nutritional Risk Screening 2002; Abx, antibiotic; HR, hazard ratio; aHR, adjusted hazard ratio; CI, confidence interval.

**Additional table 5.** Univariate and multivariate Cox regression analyses of factors associated with weaning success among septic patients.

|  | **Univariate Multivariate*** | | | | |
| --- | --- | --- | --- | --- | --- |
|  | **Crude HR (95% CI)** | ***P*** |  | **Adjusted HR (95% CI)** | ***P*** |
| Plasma TMAO |  |  |  |  |  |
| High (≥2.5 μmol/L) | Ref | Ref |  | Ref | Ref |
| Median (0.4-2.5 μmol/L) | 0.52 (0.23-1.21) | 0.131 |  | 0.62 (0.26-1.46) | 0.272 |
| Low (<0.4 μmol/L) | 0.23 (0.08-0.65) | 0.005 |  | 0.32 (0.11-0.91) | 0.033 |
| Age | 1.01 (0.98-1.04) | 0.436 |  |  |  |
| Male gender | 0.92 (0.43-1.94) | 0.822 |  |  |  |
| Smoker | 1.53 (0.65-3.61) | 0.330 |  |  |  |
| APACHE II scores | 0.93 (0.88-0.98) | 0.011 |  | 0.95 (0.90-1.00) | 0.053 |
| SOFA scores | 0.87 (0.77-0.98) | 0.024 |  |  |  |
| Mean arterial pressure | 1.01 (0.98-1.04) | 0.442 |  |  |  |
| Septic shock | 1.28 (0.60-2.75) | 0.523 |  |  |  |
| Respiratory tract infection | 0.79 (0.29-2.10) | 0.632 |  |  |  |
| Urinary tract infection | 1.12 (0.27-4.73) | 0.877 |  |  |  |
| Intra-abdominal infection | 1.32 (0.53-3.25) | 0.552 |  |  |  |
| Bloodstream infection | 0.79 (0.38-1.68) | 0.547 |  |  |  |
| Antibiotic pre-treatment, numbers | 0.83 (0.68-1.02) | 0.076 |  |  |  |
| Antibiotic pre-treatment, days | 0.96 (0.91-1.00) | 0.066 |  |  |  |
| Hypertension | 1.45 (0.69-3.04) | 0.330 |  |  |  |
| Diabetic mellitus | 0.76 (0.31-1.88) | 0.552 |  |  |  |
| Heart failure | 1.16 (0.35-3.87) | 0.805 |  |  |  |
| COPD | 1.01 (0.24-4.25) | 0.991 |  |  |  |
| Cirrhosis | 1.49 (0.35-6.30) | 0.587 |  |  |  |
| Prior CAD | 1.43 (0.43-4.76) | 0.563 |  |  |  |
| Prior stroke or PAD | 1.27 (0.38-4.23) | 0.693 |  |  |  |
| Malignancy | 0.57 (0.27-1.22) | 0.150 |  |  |  |
| Autoimmune disease | 1.12 (0.39-3.23) | 0.837 |  |  |  |
| White blood cells (K) | 1.01 (0.97-1.06) | 0.508 |  |  |  |
| Hemoglobin | 1.07 (0.91-1.28) | 0.407 |  |  |  |
| eGFR at ICU admission | 0.99 (0.98-1.00) | 0.057 |  |  |  |
| Total bilirubin | 0.94 (0.82-1.08) | 0.384 |  |  |  |
| Glucose | 1.00 (1.00-1.01) | 0.400 |  |  |  |
| Lactate | 1.00 (0.99-1.02) | 0.873 |  |  |  |
| C-reactive protein | 1.04 (1.01-1.08) | 0.013 |  | 1.04 (1.01-1.08) | 0.024 |
| Enteral intake of calories | 1.00 (1.00-1.00) | 0.045 |  |  |  |
| Enteral intake of protein | 1.01 (1.00-1.02) | 0.068 |  |  |  |
| Total calories (% of target) | 1.01 (1.00-1.01) | 0.258 |  |  |  |
| Total protein (% of target) | 1.01 (1.00-1.02) | 0.051 |  |  |  |
| Body mass index | 1.05 (0.99-1.12) | 0.124 |  |  |  |
| Albumin | 1.79 (0.92-3.49) | 0.086 |  |  |  |
| Prealbumin | 1.07 (1.10-1.13) | 0.038 |  |  |  |
| Nutrition risk index | 1.03 (1.00-1.06) | 0.023 |  |  |  |
| NRS 2002 scores | 0.74 (0.51-1.08) | 0.115 |  |  |  |

*Adjusted for variables with *p*<0.1 in the univariate analysis.

HR, hazard ratio; CI, confidence interval; TMAO, trimethylamine-N-oxide; Ref, reference; APACHE, Acute Physiology and Chronic Health Evaluation; SOFA, Sequential Organ Failure Assessment; CAD, coronary artery disease; CI, confidence interval; COPD, chronic obstructive pulmonary disease; CAD, coronary artery disease; PAD, peripheral arterial disease; eGFR, estimated glomerular filtration rate; HR, hazard ratio; NRS- 2002, Nutritional Risk Screening 2002.
